# Transcellular blood–brain barrier disruption in malaria-induced reversible brain edema

**DOI:** 10.26508/lsa.202201402

**Published:** 2022-03-08

**Authors:** Jessica Jin, Mame Aida Ba, Chi Ho Wai, Sanjib Mohanty, Praveen K Sahu, Rajyabardhan Pattnaik, Lukas Pirpamer, Manuel Fischer, Sabine Heiland, Michael Lanzer, Friedrich Frischknecht, Ann-Kristin Mueller, Johannes Pfeil, Megharay Majhi, Marek Cyrklaff, Samuel C Wassmer, Martin Bendszus, Angelika Hoffmann

**Affiliations:** 1 Department of Neuroradiology, Heidelberg University Hospital, Heidelberg, Germany; 2 Division of Experimental Radiology, Department of Neuroradiology, Heidelberg University Hospital, Heidelberg, Germany; 3 Centre for Infectious Diseases, Parasitology Unit, Heidelberg University Hospital, Heidelberg, Germany; 4 Center for the Study of Complex Malaria in India, Ispat General Hospital, Rourkela, India; 5 Department of Intensive Care, Ispat General Hospital, Rourkela, India; 6 Department of Neurology, Division of Neurogeriatrics, Medical University of Graz, Graz, Austria; 7 German Center for Infection Research (DZIF), Heidelberg, Germany; 8 Center for Childhood and Adolescent Medicine, General Pediatrics, University Hospital, Heidelberg, Germany; 9 Department of Radiology, Ispat General Hospital, Rourkela, India; 10 Department of Infection Biology, London School of Hygiene and Tropical Medicine, London, UK; 11 University Institute of Diagnostic and Interventional Neuroradiology, University Hospital Bern, Inselspital, University of Bern, Bern, Switzerland

## Abstract

Brain swelling occurs in cerebral malaria (CM) and may either reverse or result in fatal outcome. It is currently unknown how brain swelling in CM reverses, as brain swelling at the acute stage is difficult to study in humans and animal models with reliable induction of reversible edema are not known. In this study, we show that reversible brain swelling in experimental murine CM can be induced reliably after single vaccination with radiation-attenuated sporozoites as proven by in vivo high-field magnetic resonance imaging. Our results provide evidence that brain swelling results from transcellular blood–brain barrier disruption (BBBD), as revealed by electron microscopy. This mechanism enables reversal of brain swelling but does not prevent persistent focal brain damage, evidenced by microhemorrhages, in areas of most severe BBBD. In adult CM patients magnetic resonance imaging demonstrate microhemorrhages in more than one third of patients with reversible edema, emphasizing similarities of the experimental model and human disease. Our data suggest that targeting transcellular BBBD may represent a promising adjunct therapeutic approach to reduce edema and may improve neurological outcome.

## Abstract (precis)

We present how reversible edema can reliably be induced in experimental cerebral malaria and show that it is associated with transcellular blood–brain barrier disruption and delayed microhemorrhages.

## Introduction

Cerebral malaria (CM) is the most severe complication of *Plasmodium falciparum* infection. It is characterized by altered consciousness and coma, and by brain swelling in children and, to a lesser degree, in adults. Mortality is high, despite adequate anti-parasitic treatment, and long-term neurocognitive impairment are reported in about one third of surviving patients (1, 2, 3).

A hallmark of CM is the sequestration of parasite-infected erythrocytes in the microvasculature, a process described in both human and experimental CM (1, 4, 5). Pathological mechanisms in CM have been related to endothelial dysfunction and increased blood–brain barrier disruption (BBBD) and it is well accepted that this event precedes vasogenic brain edema (6, 7). BBBD has long been considered to be caused by disrupted tight junctions (TJs) between endothelial cells in vessels, a phenomenon also called paracellular BBBD (8). However, a report on stroke (9), which is also associated with reversible brain edema, suggests a transcellular BBBD as the main mechanism of early edema formation in stroke. It remains unclear whether transcellular BBBD occurs in other diseases with reversible brain swelling, and which factors determine the reversibility of brain swelling.

Despite reversal of brain swelling, structural changes such as microhemorrhages, have been reported in survivors of CM (10, 11). However, it remains unclear how and at what stage of the disease microhemorrhages occur. Reversibility of vasogenic brain edema and the occurrence of microhemorrhages can be assessed in vivo by magnetic resonance imaging (MRI), but further invasive analyses in humans are currently limited. In addition, pathophysiological studies have been hampered by the lack of animal models that show reversible edema reliably. Investigating the mechanisms underlying reversible edema is necessary to establish new therapeutic approaches and to eventually reduce permanent brain damage in CM. An animal model with reliable induction of reversible edema would thus represent a valuable tool to gain insight into these pathogenetic events.

Using serial in vivo MRI, we demonstrated for the first time the reproducible induction of reversible brain edema in a murine model of CM. We identified transcellular BBBD as an edema mechanism and showed that microhemorrhagic remnants occur in areas of higher BBBD. We further highlighted the translational potential of this experimental model by illustrating similar patterns of microhemorrhages in experimental cerebral malaria (ECM) and in human CM.

## Results

### Reversible brain swelling can be reliably induced in ECM

**Figure 1.**
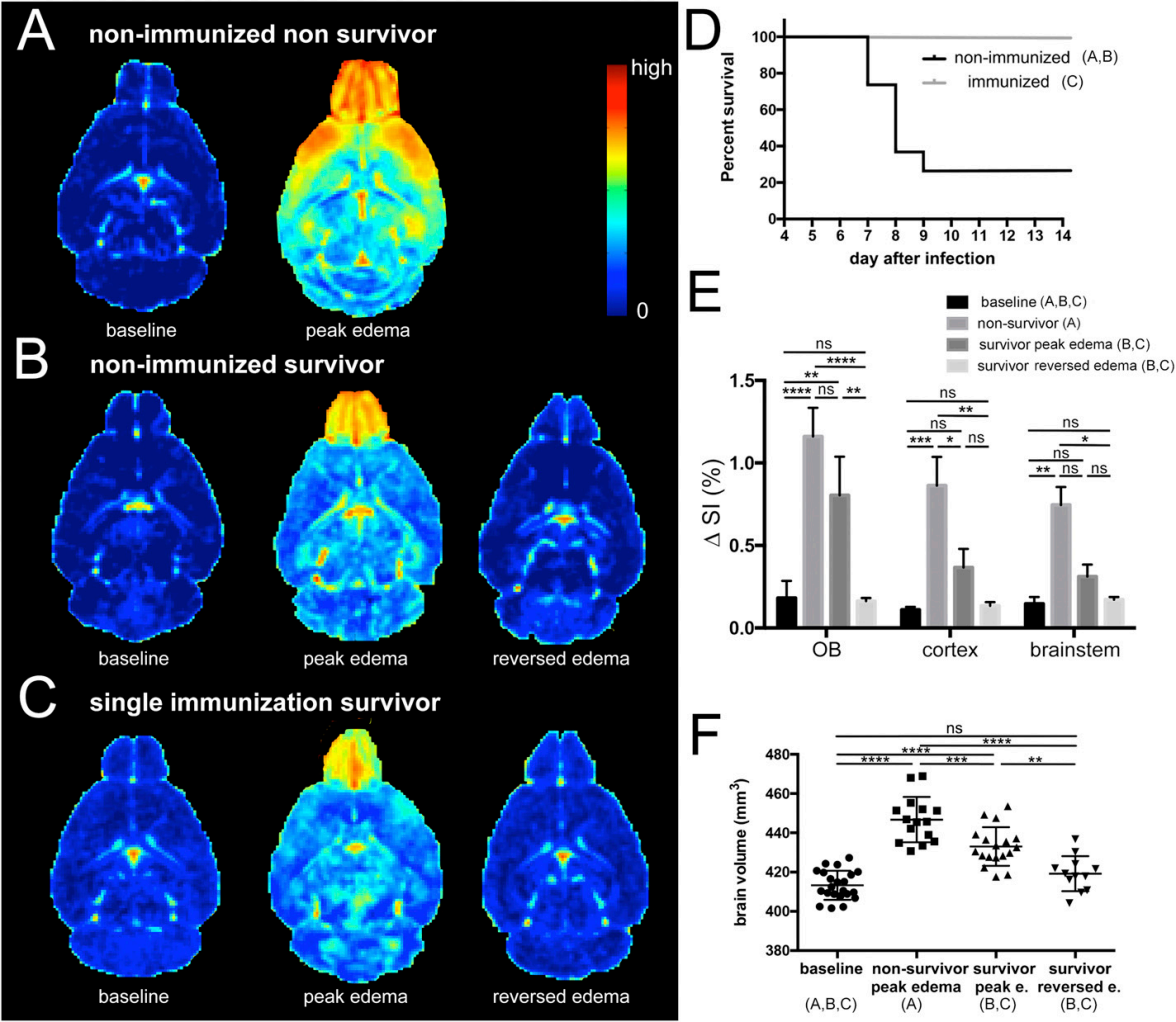
Reversible brain swelling in experimental cerebral malaria. **(A, B, C)** Subtraction images of T1-weighted images are displayed to illustrate the degree and spatial distribution of blood–brain barrier disruption (BBBD) in surviving and non-surviving mice. **(D)** Survival curves of wild-type infected mice and mice after single vaccination (A n = 15, B n = 5; C n = 13). **(E)** The difference in signal intensity (ΔSI) as a measure of BBBD is present in the olfactory bulb, cortex and brainstem in all groups. Most pronounced signal alterations are seen in the olfactory bulb. In cortex and brainstem less BBBD is seen in survivors. After edema resolves BBB, normalizes and returns to baseline values. **(F)** The degree of brain swelling is shown at baseline, in non-survivors and survivors at the acute stage and after edema has reversed in surviving mice. Peak edema occurred at day 8.1 ± 0.6 d post infection in non-immunized non-survivors, at day 8.8 ± 0.4 d post infection in non-immunized survivors and at 9.4 ± 0.8 d post infection. Edema lasted 1–3 d. Day 11 after infection was the last day edema was observed. Day 14 after infection was the imaging time point for reversed edema in all mice. Significance levels were tested with one-way ANOVA.

To test whether the ECM model associates with reversible brain swelling, we performed serial MRI on C57Bl/6 mice infected with a low number (1,000) of *Plasmodium berghei* (Pb ANKA) sporozoites. We examined BBBD and edema evolvement in all mice as soon as the first animal developed ECM and followed further progress of disease that led to either recovery or death. Non-surviving mice showed imaging characteristics on MRI that we previously described, including BBBD with rostral predominance with peak edema at day 8.1 ± 0.6 d post infection (Fig 1A) (12). Notably, 5 of 20 mice survived. These surviving mice also exhibited BBBD, albeit less pronounced with peak edema at day 8.8 ± 0.4 d post infection (Fig 1B). It is noteworthy that similar MRI findings have been demonstrated in 55% of *Pb* ANKA–infected mice treated with a glutamine antagonist (13). However, the lack of predictive markers to identify edema reversibility at the acute stage of disease has hampered investigation of pathogenetic mechanisms associated with reversible versus irreversible edema. To circumvent this limitation, we set out to develop a model in which reversible edema could be reliably induced. We reasoned that partial protection of mice could be induced by immunization with attenuated sporozoites. A previous study demonstrated complete protection from ECM after single vaccination, with 100% survival, but no sterile protection against malaria infection (14). We therefore investigated whether reversible edema occurred in mice after single vaccination with radiation-attenuated sporozoites (RAS). In our set up, all 20 wild-type mice after a single vaccination with RAS survived a subsequent challenge with sporozoites. 13 of these mice (65%) showed reversible BBBD with peak edema at 9.4 ± 0.8 d post infection (Fig 1C). The remaining seven vaccinated mice did not show alterations in brain on MRI signal (five of these developed parasitemia). In comparison, 25% (5 out of 20) of non-immunized wild-type mice survived after sporozoite challenge with a low number of sporozoites (Fig 1D). Thus, a single vaccination with RAS is protective against fatal ECM but infected mice often develop reversible edema, a disease pattern which is observed to a lower rate after infection of non-immunized wildtype mice.

**Figure S1.**
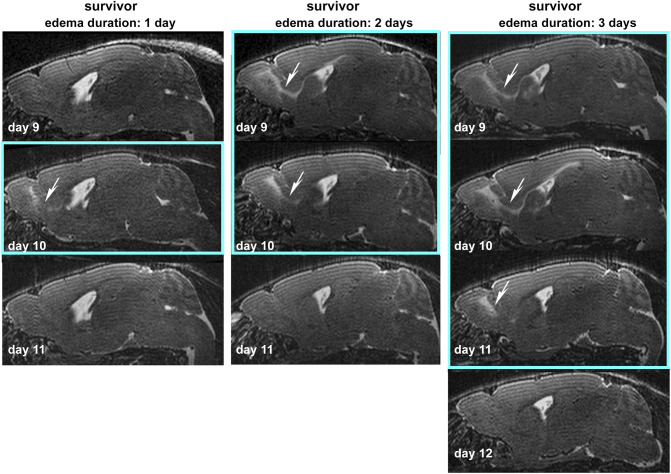
Edema duration. Exemplary T2w sagittal images of three surviving mice are displayed. Turquoise squares surround images with edema. Edema (white arrows), visible as increased (bright) T2w signal was evident for 3 d, if it had reached the dorsal migratory stream, or lasted for 1–2 d if mainly the olfactory bulb and rostral migratory stream were affected.

**Figure S2.**
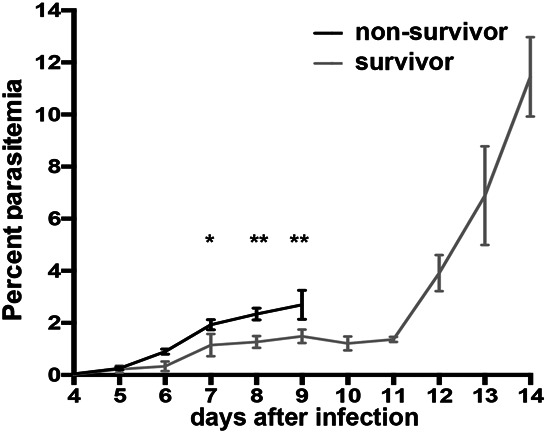
Parasitemia course. Parasitemia increases slower in surviving mice and is significantly different to non-survivors from day 7 to day 9. * indicates significance levels as calculated by the unpaired, two tailed *t* test.

At peak edema wild-type non-immunized survivors and single immunized mice with edema showed significantly less BBBD and significantly less brain volume increase compared with non-surviving mice (Fig 1E and F). No significant differences were seen between wild-type non-immunized survivors and single immunized mice. Overall, edema lasted 1–3 d before it reversed (Fig S1) and parasitemia increased at a slower rate in surviving mice (wildtype non-immunized and single immunized mice) compared with non-surviving mice (Fig S2). Survivors exhibited slower parasite growth rates and less fulminant edema, confirming that priming of the immune system and host-controlled parasite growth by single vaccination provides protection from severe disease (14, 15, 16). At day 14, the last imaging time point, brain swelling had reversed and returned to baseline volumes (Fig 1F).

### Transcellular BBBD mediates reversible brain edema

**Figure 2.**
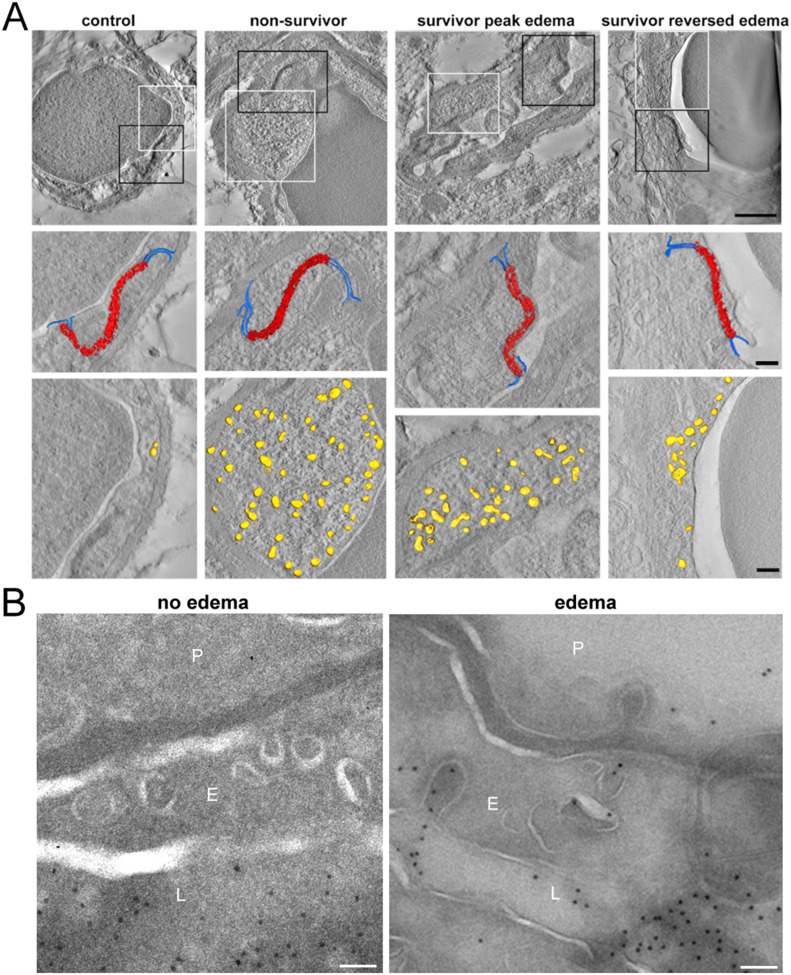
Reversible edema is induced by transcellular blood brain barrier disruption. **(A)** Tomographic slices of example vessels are displayed with 3D models projected on the slice. Black squares are magnified below to demonstrate intact tight junctions (red) and adjacent adherence junctions (blue). White squares are magnified below and illustrate the increased number of vesicles in the endothelium (yellow), occurring during transcellular blood–brain barrier disruption. Scale bar first row 1 μm, scale bar second and third rows 200 nm. **(B)** DNP-Gold labelled Tokuyasu sections of mice injected with DNP-albumin. In a control mouse without edema, DNP-albumin remains intraluminal (first images). In an experimental cerebral malaria mouse with edema, DNP-albumin reaches the parenchymal side via vesicles (second image). L = lumen, E = endothelium, P = parenchyma. Scale bar 100 nm.

**Figure S3.**
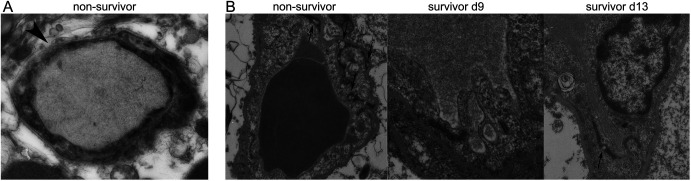
Basal lamina and tight junctions. **(A)** Dissolvement of the basal lamina in an example vessel from a non-surviving mouse is shown (arrowhead). **(B)** Abnormal tight junction with gaps (arrows) are seen in non-survivors and rarely in mice after edema had reversed, but not in surviving mice at peak edema. Scale bar 200 nm.

The single RAS-immunization model allowed us to study not only cerebral changes after edema reversal, but also for the first time its acute stage. We therefore addressed the mechanisms leading to the development of BBBD and its reversal by analyzing ultrastructural changes underlying these processes. We focused on the endothelium of small brain vessels using electron microscopy, which allows the discrimination between paracellular and transcellular BBBD (9). Whereas the former is triggered by ruptured TJs between endothelial cells, the latter is characterized by an increase in intra-endothelial vesicles transporting fluid from the luminal side to the brain parenchyma. In healthy control mice, intact TJs and few intracellular vesicles were apparent, both characteristics of an intact BBB (Fig 2A). In non-survivors, 70% of TJs remained intact, whereas the number of intra-endothelial vesicles increased by 1,600% compared with healthy controls (Fig 2A). Dissolution of the basal lamina was also seen (Fig S3A and B). In survivors at the acute stage of edema, TJs and basal lamina remained fully intact, whereas intracellular vesicles increased by 1,100% compared with healthy controls (Figs 2A and S3B). Upon the reversal of edema, the number of vesicles decreased by almost a half to 700% of the healthy control level, whereas 97% of TJs remained intact (Fig 2A), and disrupted TJs were seen in 3% of acquired images. No dissolution of the basal lamina was observed (Fig S3B). To test if vesicles serve as vehicle for inflammatory agents, for example, fluid and proteins to reach the parenchymal side, we injected DNP-albumin, and visualized it with immunogold particles (Fig 2B). In healthy mice with no edema most DNP-albumin remained intraluminal, whereas it was visible within vesicles and on the parenchymal side in mice with edema (Fig 2B).

### Microvascular damage occurs after edema reversal

**Figure 3.**
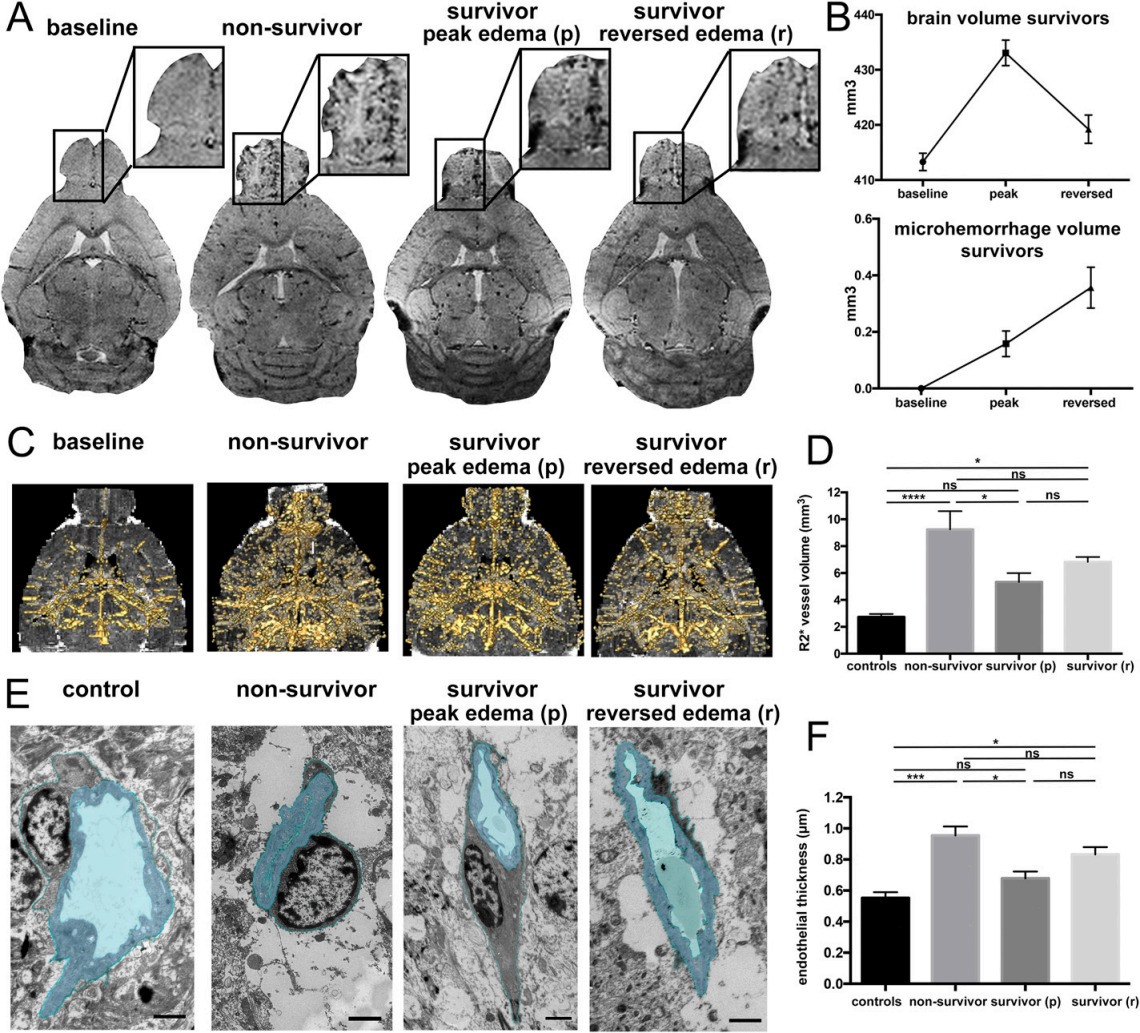
Reperfusion injury on the microscopic and ultrastructural level. **(A)** Exemplary T2*w images are displayed. At baseline, no microhemorrhages are apparent (arrows). In severe disease a high microhemorrhage load is seen, mainly in the olfactory bulb (OB). In survivors microhemorrhages increase after edema has resolved. **(B)** Graphs illustrate the increase of microhemorrhage volume, whereas brain volume reverses. **(C)** Segmented vessels (gold) on R2* datasets are displayed. The segmentation delineates vessel lumen and vessel wall. In non-survivors vessel volume is highest. **(D)** R2* vessel volume throughout the groups is presented. (n = 5–13). **(E)** Ultrastructural endothelial changes on TEM images are illustrated by one example of a vessel from each group. In healthy mice, the endothelium is thin with an open lumen. Astrocyte end feet cover the endothelium. In non-survivors, the endothelium is thicker, the lumen collapsed, and surrounded by swollen astrocyte end feet. Survivors in the acute stage of disease show a slight increase in endothelial thickness, but also swollen astrocyte end feet. Astrocyte end feet swelling decreases after edema has reversed, but endothelial thickness slightly increases. **(F)** Average endothelial thickness in all groups is displayed (n = 3–5); Significance levels were tested with one-way ANOVA.

Microhemorrhages were visible by MRI in areas of most severe BBBD. These were predominantly located in the olfactory bulb and were more numerous in non-survivors (Fig 3A). In survivors at peak of disease, significantly less microhemorrhages were apparent, compared with non-survivors. Even though BBBD and edema reversed in survivors, microhemorrhages remained visible and surprisingly significantly increased after edema had reversed (Fig 3A and B).

**Figure 4.**
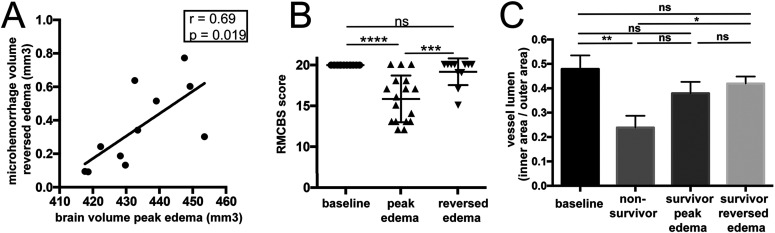
Correlation and time course of reperfusion injury and behavioral changes. **(A)** Microhemorrhage volume correlates with the degree of brain swelling at the acute stage of the disease. **(B)** Behavioral changes were assessed with the Rapid Murine Coma and Behavioral Scale (RMCBS) score. At baseline all mice displayed a healthy score of 20. Survivors showed altered behavior during the acute stage of disease but returned back to normal in most mice. **(C)** The ratio of inner area of the endothelium versus the outer area of the endothelium is displayed as a measure of collapse of lumen. Significance levels were tested with one-way ANOVA. Spearman’s analyzes were used for correlation analyses.

Interestingly, surviving mice that exhibited larger brain volumes in the acute stage of disease, developed significantly more microhemorrhages after edema had resolved, showing that initial disease severity correlates with the degree of microhemorrhages after recovery (Fig 4A).

To investigate the association between imaging features and behavioral alterations, the rapid murine coma and behavioral scale (RMCBS) score was used. 30% of surviving mice did not reach a healthy baseline score of 20 after edema had resolved. The remaining 70% reached a baseline score of 20 despite exhibiting microhemorrhages (Fig 4B). To further analyze if the vasculature itself shows impairment, we examined the vasculature in affected brain volumes at microscopic and ultrastructural levels. On MRI-derived R2* maps, which quantify susceptibility signal of deoxygenized blood on MR images, we assessed the vascular volume in vivo over time at a resolution of 50 μm. Vessel volume within R2* maps in survivors showed a trend towards a vessel volume increase when edema had reversed (Fig 3C and D). Corroborating that injurious processes continue developing further, we observed endothelial alterations at the ultrastructural level matching the observed in vivo changes detected by R2* maps. Even though vessel lumen normalized after edema had reversed (Fig 4C), the endothelial perimeter increased in survivors, indicating endothelial remodeling and consecutive increased vascular reactivity that promotes peripheral resistance (Fig 3E and F). Altogether, these findings provide evidence that microvascular alterations occur after edema reversal.

### Comparison of MRI findings in human disease and the experimental model

**Figure 5.**
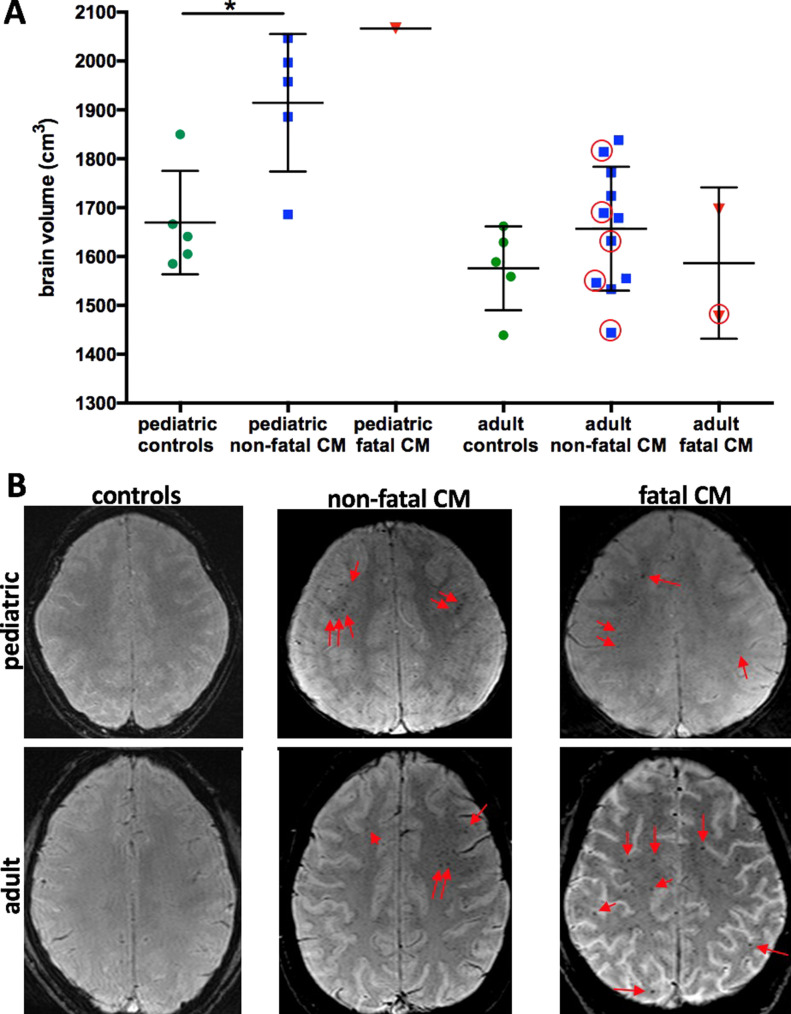
Brain swelling and occurrence of microhemorrhages in pediatric and adult cerebral malaria (CM). **(A)** Normalized brain volume in children was significantly increased compared with age-matched controls and more pronounced in children compared with adults. In adults, no significant increase compared with healthy volunteers was noted, despite a spread in brain volume in non-fatal CM and fatal CM. Normalized brain volumes could only be calculated in datasets without motion artefacts. Red circles label data points of patients with successful brain volume calculation and presence of microhemorrhages. In this cross-sectional dataset, brain volumes of patients with microhemorrhages show a wide range. Significance levels were tested with one-way ANOVA. **(B)** Exemplary susceptibility-weighted images are shown. In controls no microhemorrhages are visible. In non-fatal and fatal disease red arrows point to exemplary microhemorrhages at the subcortical white matter.

To investigate the relevance of our model to human CM, we compared brain volume measurements and the occurrence of microhemorrhages of human and experimental findings. Human MR datasets were analyzed from both pediatric and adult Indian CM patients admitted at Ispat General Hospital in Rourkela, India, as part of a study described elsewhere (17, 18), which included susceptibility-weighted imaging (SWI), a sensitive MRI sequence to detect microhemorrhages. In this subgroup, similar distributions of brain volume were apparent as previously published with higher normalized brain volumes in pediatric CM compared with adult CM (Fig 5A) (18). The experimental model uses young mice and also shows high brain volumes, similar to pediatric CM patients. CM patients with microhemorrhages showed a wide range of brain volumes (Fig 5A). As only one SWI dataset was acquired in most patients, we could not assess the temporal course of microhemorrhage occurrence and cannot prove if there is a delayed occurrence of microhemorrhages in patients. The presence of microhemorrhages did not differ in adult and pediatric CM, with 50% in fatal CM (one of two pediatric CM patients as well as one of two CM adult patients), 33% in non-fatal pediatric CM (three of nine patients), and 36% in non-fatal adult CM (5 out of 14 patients) (Table S1). Similar to the experimental model, microhemorrhages are more frequent in fatal compared with non-fatal disease during the acute stage of the disease. The anatomic predilection site of microhemorrhages in the brain was not the olfactory bulb but the grey and white matter junction (Fig 5B), as well as the corpus callosum, basal ganglia, and cerebellum. Despite different microhemorrhage location in the mouse model and human CM, microhemorrhages occur during both pediatric and adult CM. As microhemorrhages are also present in patients with lower brain volumes, they may have occurred after edema had reversed. Further studies on the temporal course of microhemorrhage occurrence will be able to answer fully, if microhemorrhages also occur in a delayed fashion in CM patients.

Table S1 Number of CM patients with microhemorrhages.

## Discussion

In this study, we demonstrated that (i) transient, reversible brain swelling can reliably be induced in ECM, (ii) that transcellular BBBD is an important mechanism of edema development in ECM and (iii) that permanent damage remains in areas of most severe BBBD. We further highlighted the translational potential of the model by comparing the experimental model with clinical findings.

Using a model of single-immunization with irradiated sporozoites allowed us to investigate for the first time the pathogenic events during the acute phase of the disease leading to reversible vasogenic edema. We emphasize that MRI is necessary to select immunized mice that develop edema. However, in contrast to wild-type mice that sometimes also show reversible edema, edema in single immunized mice is reversible in 100% of cases. In wildtype mice, or other models with medication-induced reversible edema, it is not possible to predict at the acute stage of the disease, if they will survive or not. Thus, the single-immunization model offers a model to reliably study edema at reversal, a CM-specific feature that is not possible to study in humans, apart from imaging studies.

We identified transcellular BBBD without disrupted TJs as a mechanism of brain swelling in ECM. Up-regulation of intra-endothelial vesicles is the initial response of brain endothelium to ischemia/reperfusion and to inflammation but has been largely overlooked as a step of early BBBD (9, 19, 20, 21). Although the number of intra-endothelial vesicles increased, intact TJs indicate that endothelial cells keep their lining, without pulling TJ complexes apart or being destroyed by proteinases, thus enabling edema reversal (22). This is in line with our observations in fatal ECM, as well as previous findings in both ECM and CM, showing disrupted TJs and higher numbers of intra-endothelial vesicles associated with irreversible edema (5, 23, 24, 25). By analyzing both survivors and non-survivors for the first time and by performing tracer experiments, we now extend previous reports and identify endothelial vesicles and thus transcellular BBBD as a contributing mechanism to brain swelling in our model of ECM. In other diseases such as stroke, transcellular BBBD precedes TJ disruption, which occurs in later phases of BBBD and represents potentially more severe damage (9, 26). It is therefore conceivable that transcellular BBBD is not only responsible for reversible vasogenic edema in CM but may also be involved in reversible brain edema in general.

Despite normalization of paracellular BBBD and edema reversal, a delayed occurrence of microhemorrhages and vascular remodeling was observed in animals that survived ECM. Small hemorrhages have been linked to severe transcellular BBBD after reperfusion in an experimental stroke model (27). In our study, microhemorrhages mainly occurred in a delayed fashion in the olfactory bulb, the region of most pronounced BBBD during the acute disease stage. Thus, in the region with most severe endothelial damage at the acute stage of the disease further damage was observed after brain swelling had reversed and blood flow normalized. There are several explanations for those observations, including that impaired vessels may not withstand the increased pressure, thereby leading to microhemorrhages or that continued extravasation through damaged vessels causes the delayed occurrence of microhemorrhages.

Remarkably, in a model of hypoxia/reperfusion, which mimics high-altitude cerebral edema, mice exhibited a similar temporal course of microhemorrhages with a strong increase after reoxygenation in the same anatomic areas (28). The location of microhemorrhages in human CM differed from the experimental model and the microhemorrhages are seen predominantly in the corpus callosum, the subcortical white matter, and the basal ganglia. However, they developed at the same locations as in other human disease with hypoxia/reperfusion, such as high-altitude edema (29, 30). These similarities suggest that (i) hypoxia is an important factor in ECM/CM pathogenesis; (ii) reperfusion and reoxygenation may lead to microhemorrhages; and (iii) they indicate prior vascular damage during acute disease. The importance of hypoxia during ECM pathogenesis and during CM has been described previously (18, 31, 32), and the observed endothelial swelling may also be induced by hypoxia (33).

We expand these findings and further demonstrate that reperfusion injury occurs in a similar fashion than in other diseases that involve hypoxia/reperfusion. Interestingly, a review article about CM from 1997 postulated that microhemorrhages may be induced by reperfusion injury due to their distinct pathological features (34). Indeed, the unregulated restoration of flow in a damaged cerebral microvessel previously filled with sequestered parasitized red blood cells and host monocytes may cause its rupture, forcing the contents out into the brain parenchyma, and a subsequent leakage of non-parasitized erythrocytes (34).

Because we show that microhemorrhages in ECM survivors occur when brain swelling reverses, microhemorrhages likely mark areas that are most severely damaged during acute disease. These cerebral microhemorrhagic “scars” can also be detected by MRI on long-term follow-up scans in diseases with reversible brain swelling (35). The number and intensity of microhemorrhages warrants further investigation in CM patients as they may indicate initial disease severity in CM. It is noteworthy that microhemorrhages may not be clinically silent. They have been associated with long-term neurological impairment in patients in several diseases, including diffuse axonal injury, small vessel disease, and cognitive decline in dementia, and may represent markers for the degree of neurocognitive impairment (36, 37, 38). Neurological sequelae are known to occur after CM (3) and could potentially be linked to microhemorrhages. They may thus represent a powerful marker to predict potential neurocognitive impairment and warrant further clinical investigation.

Taken together, our results suggest a potential association between the degree of initial transcellular BBBD, consecutive brain swelling and reperfusion injury. This association indicates that adjuvant drug treatment targeting transcytosis could reduce reperfusion injury and protect from long-term neurocognitive impairment.

Drugs suppressing transcytosis in brain endothelial cells such as the protein kinase inhibitor imatinib have been shown to improve outcomes in ECM, as well as in experimental and human stroke (39, 40, 41, 42, 43). They are thus capable of effectively reducing edema caused by hypoxic BBBD. Inhibiting endothelial transcytosis of the BBB by enhancing the major facilitator superfamily domain-containing protein 2 (Mfsd2a), for example, may also improve outcomes in disease with hypoxic BBBD (44, 45). Either already approved drugs or new drugs targeting transcellular BBBD and not paracellular BBBD may therefore offer a widely applicable treatment option in diseases inducing reversible edema and merit further investigation.

## Materials and Methods

### Ethics statement

#### Mouse model

All animal experiments were performed according to FELASA category B and ARRIVE guidelines and approved by the local German authorities in Karlsruhe.

#### Patients

Ethical approval was obtained from The Indian Council of Medical Research as well as from the institutional review boards of Ispat General Hospital, New York University School of Medicine and the London School of Hygiene and Tropical Medicine. Because CM patients were comatose, written informed consent was obtained from the families of all patients before enrollment in the study as previously described (17, 18).

### Murine malaria model

ECM was induced with the *P. berghei* ANKA (*Pb* ANKA) parasite in inbred female C57BL/6J mice (Janvier Labs). *Pb* ANKA sporozoites (SPZ) were isolated by dissection of salivary glands from female *Anopheles stephensi* mosquitoes at day 18–21 post infection. Infections of 6–8-wk-old female mice were performed by i.v. injections of 1,000 SPZ in a total volume of 100 μl sterile PBS (n = 12). In a second group the same infections in 12–14-wk-old mice were carried out in naïve mice (n = 8) and single vaccinated mice (n = 20) (14). For immunization with RAS, SPZ were treated by exposure to 150 Gy of γ-radiation (^137^Cesium source, University Hospital Heidelberg) and were then injected into mice at a dose of 3 × 10^4^ RAS. Brains of three healthy female C57BL/6J mice were used as controls for tissue analysis. Parasitemia was assessed starting at day 4 after infection.

For clinical evaluation, malaria-infected mice were assessed for 10 parameters of cerebral symptoms according to the rapid murine coma and behavioral scale (RMCBS) (46). The RMCBS testing was performed daily starting at day 6 after infection.

### Experimental MRI protocol

MRI was performed on a 9.4 T small animal scanner (BioSpec 94/20 USR; Bruker Biospin GbmH) using a volume resonator for transmission and a 4-channel-phased-array surface receiver coil. Anesthesia was induced per inhalation using 2% and maintained with 1–1.5% isoflurane. Mice were placed prone in fixed position monitoring body temperature and respiration. Starting at day 6 after infection mice were screened daily for edema until day 12 by using a 2D T2-weighted sequence (repetition time/echo time [TR/TE] = 2,000/22 ms, slices = 12, slice thickness = 0.7 mm). The baseline MR imaging protocol before infection, the MR imaging protocol at occurrence of edema (earliest detected edema occurred at day 7 after infection, the latest at day 10) and at day 14 after infection (after edema had reversed) included 3D T1-weighted imaging (TR/TE = 5/1.9 ms, flip angle [FA] = 8.5°, 156 μm isotropic resolution) before and after injection of 0.3 mmol/kg Gd-DTPA and T2*-weighted flow compensated gradient echo imaging (TR/TE = 50/18 ms, FA = 12°, 80 μm isotropic resolution) or T2* multi gradient echo imaging (TR = 50 ms, TE = 3.5–32.2 ms with increments of 5.7 ms, FA = 14°, 100 μm isotropic resolution).

### Electron microscopy of mouse tissue

For morphological analysis mice were transcardially perfused with 0.9% saline after the last MRI scan. Brains were removed and fixed in 2% glutaraldyhyde in saline overnight at +4 degrees. OB tissue was cut in cubes and postfixed with 2% glutaraldehyde + 2% paraformaldehyde in 100 mM cacodylate buffer, followed by fixation in 1% Osmium tetroxide in cacodylate buffer and contrasting with 1% uranyl acetate in water. Samples were dehydrated through immersion in a series of increasing percentage of acetone and embedded in Spurr’s resin (Serva). Sectioning was done on a Leica UC6 microtome (Leica Microsystems) and 70 nm sections were collected on formvar-coated, copper mesh grids and imaged on a JEOL JEM-1400 electron microscope (JEOL) operating at 80 kV and equipped with a 4K TemCam F416 (Tietz Video and Image Processing Systems GmBH). For tomography 350 nm thick sections were placed on formvar-coated slot grids. Tilt series over a ±60° range were recorded in a Tecnai F20 EM (FEI) operating at 200 kV using the SerialEM software package (Mastronarde 2005) on FEI Eagle 4K × 4K CCD camera at a magnification of 9.5kx resulting in 2.24 nm pixel size. Tilted images were aligned by cross-correlation procedure. The tomograms were generated by weighted-back-projection algorithm using IMOD processing packages (47).

For tracer experiments with Dinitrophenyl (DNP)–albumin conjugate (Sigma-Aldrich), 50 mg DNP-Albumin were injected i.v. after the last MRI scan at peak edema occurrence, ranging from day 7 to day 11, and circulated for 30 min. Brains were removed and fixed in 4% paraformaldehyde, 0.016% glutaraldehyde in saline overnight at +4 degrees and then cut into 150 μm sections. After a 2-h fixation step with 4% paraformaldehyde and PHEM buffer, small tissue cubes were infiltrated with gelatine of increasing concentrations (1%, 6%, and 12%). After incubation overnight in 12% gelatine, tissue cubes were incubated with 2.3M sucrose overnight and then frozen in liquid nitrogen. Sectioning was done on a Leica UC6 cryo-microtome (Leica Microsystems). Sections were immunolabelled with a rabbit anti-DNP antibody (ABnostics) at a dilution of 1:50 and in a second step with goat anti-rabbit antibody coupled to 10 nm protein A gold (CMC university medical center Utrecht) diluted 1:50.

### Image analysis of experimental data

Image processing was undertaken in Amira 5.4 (FEI, Visualization Sciences Group). Edema was graded on 3D T2-weighted images into mild (1), moderate (2) and severe (3) as previously described (12). Blood–brain barrier permeability (BBBD) was assessed by contrast-enhanced 3D gradient echo T1-w imaging. 3D non-enhanced T1w images were subtracted from contrast-enhanced T1-weighted images. In pre- and post-contrast 3D T1-weighted images, Gibbs ringing was suppressed, and signal-to-noise ratio enhanced using a 3D spatial Gaussian low-pass filter with a resulting effective isotropic resolution of 280 μm. In case of significant motion between the sequences, images were motion corrected using a custom-made MATLAB code for rigid body registration. First the difference images were evaluated for pathological enhancement by visual inspection. Second, the relative signal enhancement ΔSI (%) in different regions-of-interest (ROI) was quantified as: ΔSI (%) = [(SI_post contrast_ − SI_pre contrast_)/SI_pre contrast_ ] × 100%. ROIs were placed after anatomical delineation manually into the following structures: (1) olfactory bulb (OB)+rostral-migratory-stream (RMS), (2) dorsal migratory stream (DMS), (3) external capsule (EC), (4) cortex, (5) basal ganglia, (6) thalamus, and (7) brainstem (BS) according to the Allen Brain Atlas (48). RMS and DMS are only visible during disease, as in healthy mice they display the same signal intensity as the surrounding tissue. Therefore, ROIs were drawn at the estimated location of the structures on the scans without signal alterations in these areas. Microhemorrhage volume was semiautomatically segmented by manual region growing using a threshold-based presegmentation on T2w* datasets.

Vessel volume was semiautomatically segmented on R2* maps, which were assessed by voxel-wise monoexponential fitting of the T2* signal decay using the freely available relaxometry tool (https://github.com/neuroimaging-mug/relaxometry). TJs and endothelial vesicles were semiautomatically segmented with Amira. Endothelial measurements were performed on EM images of vessel cross sections using ImageJ (version 1.49 s) (49). Endothelial thickness was defined as a ratio of the area of vessel lumen in cross section to that of the whole vessel. Endothelial perimeter was measured along eight equally spaced lines, expanding radially from the center of the vessel lumen.

### Study site and patients

The study was carried out at Ispat General Hospital (IGH) in Rourkela, in the state of Odisha, India, from October 2013 to November 2019 (17, 18). Patients with CM that underwent SWI were included into the analysis (total n = 27, fatal n = 4, non-fatal n = 23). All CM patients satisfied a strict definition of CM according to the modified World Health Organization criteria. CM patients with coma (defined as a Glasgow coma score of 9 of 15 for adults and a Blantyre coma score of two for young children) after correction of hypoglycemia (2.2 mmol/l) and infected with *P. falciparum* (detected by rapid diagnostic test and confirmed by the presence of asexual forms of the parasite in a peripheral blood smear) fulfilled inclusion criteria. Healthy subjects served as controls. The adult controls were imaged at the same MRI at IGH. Age-matched pediatric controls were retrospectively recruited at Heidelberg University Hospital.

### Human MRI protocol and image analysis

Imaging was performed using a 1.5-Tesla (T) Siemens Symphony MRI scanner (Siemens AG). Scanning was carried out within 10 h of admission. The MRI sequences included axial T1-weighted (TE/TR = 7.7/500 ms, slice thickness = 5 mm), T2-weighted (TE/TR = 99/4,000 ms, slice thickness = 5 mm), and SWI (TE/TR = 40/50 ms, slice thickness = 2 mm, flip angle = 12 degrees). Normalized brain volume of T1-weighted images was calculated using SIENAX, which is part of the FSL toolbox (50). The occurrence of brain swelling and microhemorrhages was visually assessed on T2-weighted and susceptibility-weighted images, respectively.

### Statistics

Data are shown as mean ± SEM. Statistical analyses were performed in PRISM (GraphPad, version 7). To compare two groups, unpaired, two-tailed *t* tests were used (e.g., parasitemia non-survivor, survivor). To compare more than two experimental groups (baseline/control, non-survivor, survivor) one-way ANOVA with multiple comparisons was performed. Spearman’s analyzes were used for correlation analyses. *P*-values ≤ 0.05 were considered statistically significant.

## Data Availability

Data will be made available by the corresponding author on reasonable request.
